# Supplementary material for: Djck1α Is Required for Proper Regeneration and Maintenance of the Medial Tissues in Planarians
Source: Cells. 2023 Feb 1;12(3):473. doi: 10.3390/cells12030473 (PMC9913719; doi:10.3390/cells12030473)
Supplement: Supplementary file 1 [file cells-12-00473-s001.zip › cells-1996178-supplementary.pdf]

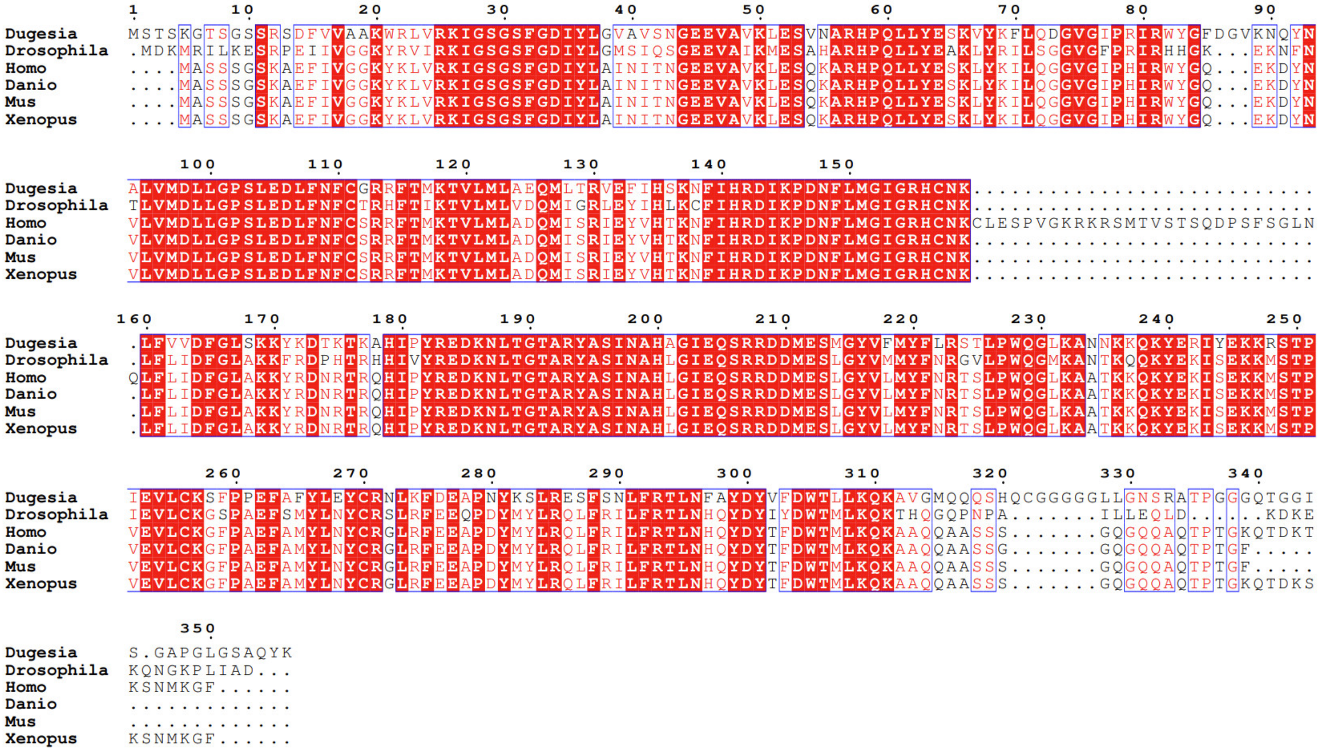

Figure S1. Multiple sequence alignment of Djck1α from *Dugesia japonica*, *Homo sapiens*, *Mus musculus, Drosophila serrata, Xenopus laevis*, and *Danio rerio.* Absolutely conserved residues are in red shaded boxes, and highly conserved residues are colored red and are boxed.

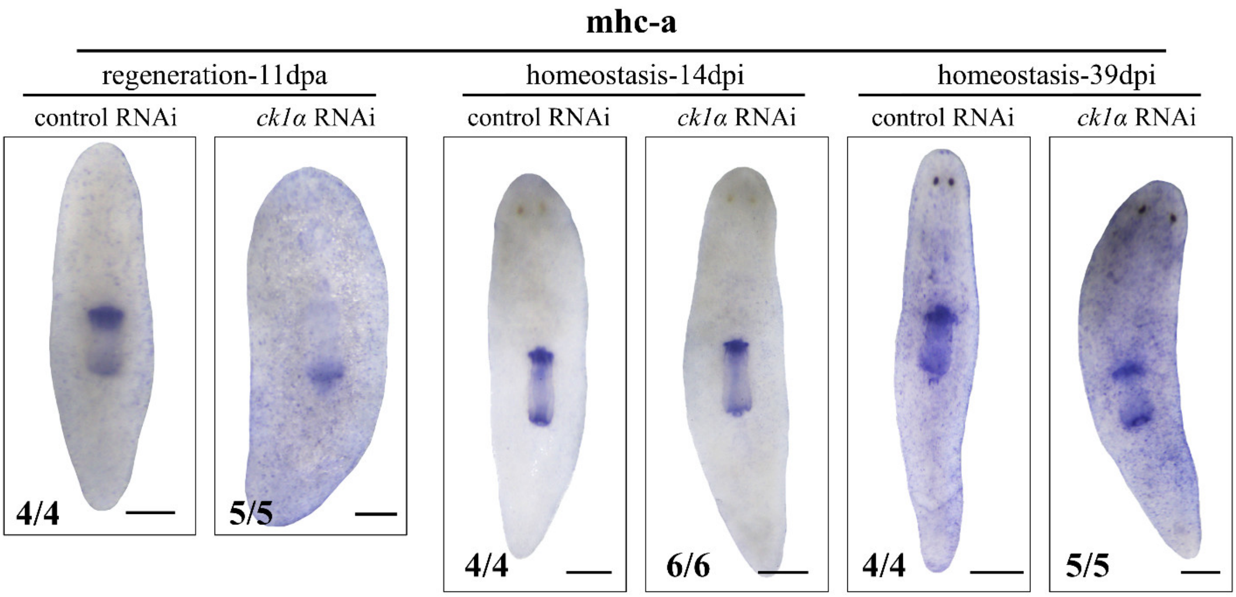

Figure S2. The WISH with Djmhc-a probes in *Djck1α* RNAi animals. Scale bars: 500 μm.

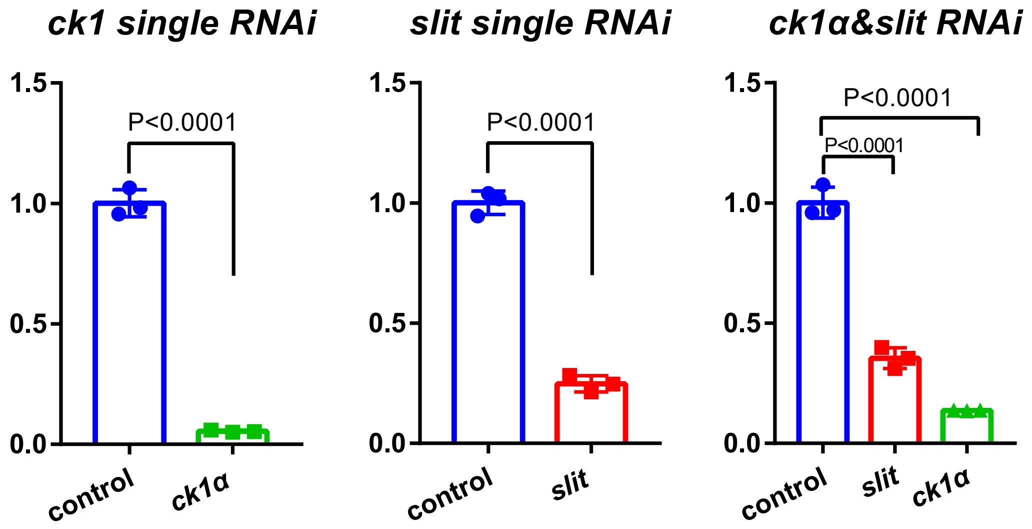

Figure S3. The down-regulation efficiency of *Djck1α* and *Djslit* in single and double RNAis animals. Left: the down-regulation efficiency of ck1α in *Djck1α* RNAi animals. Middle: the down-regulation efficiency of slit in *Djslit* RNAi animals. Right: the down-regulation efficiency of ck1α and slit in *Djck1α and Djslit* RNAi animals. unpaired Student's t-test, Mean ± SD. * $p < 0.05$.

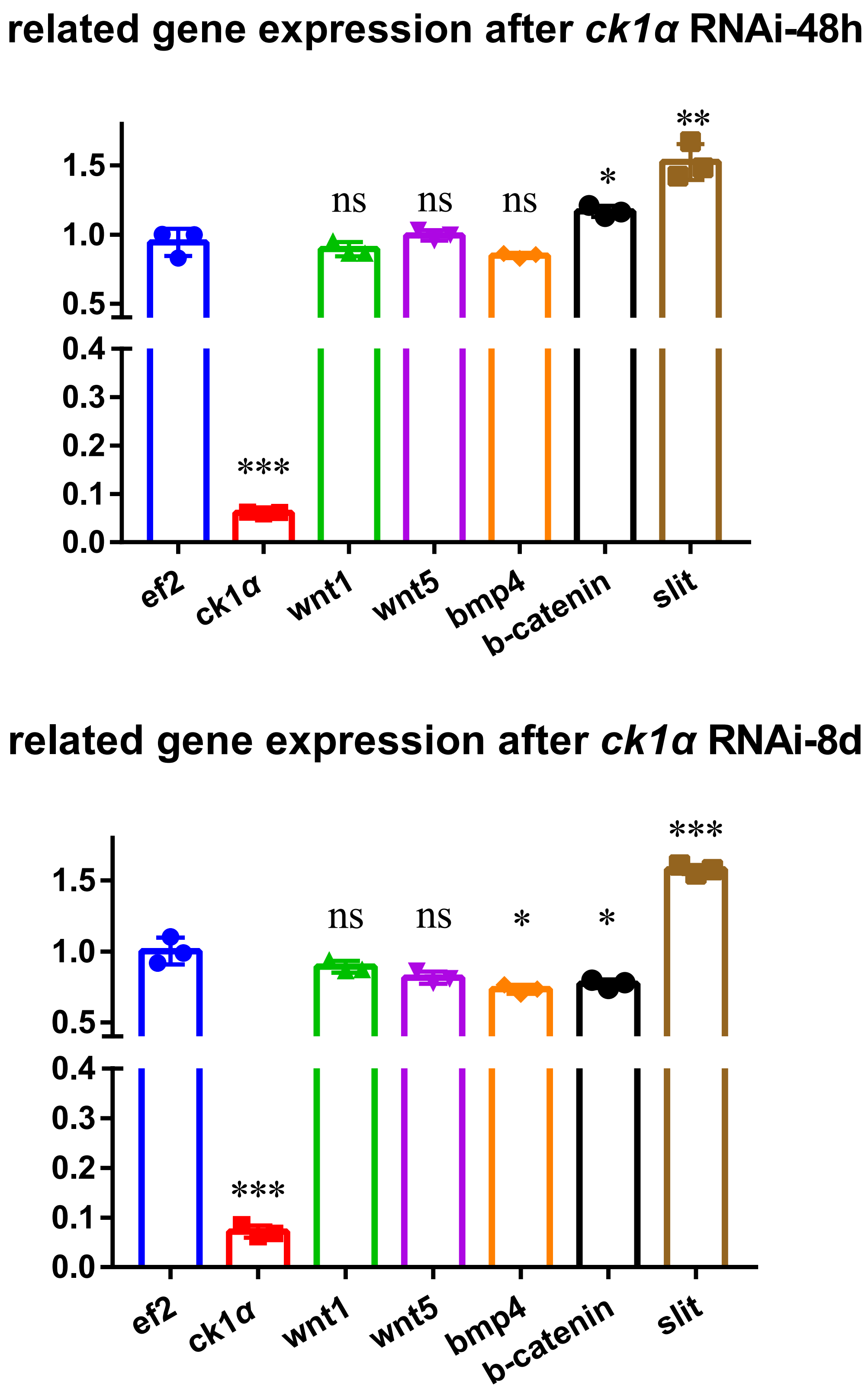

Figure S4. Related gene expression after *ck1α* RNAi. Upper: Related gene expression at 48h post the last injection. Below: Related gene expression at 8days post the last injection. unpaired Student's t-test, Mean ± SD. * $p < 0.05$.

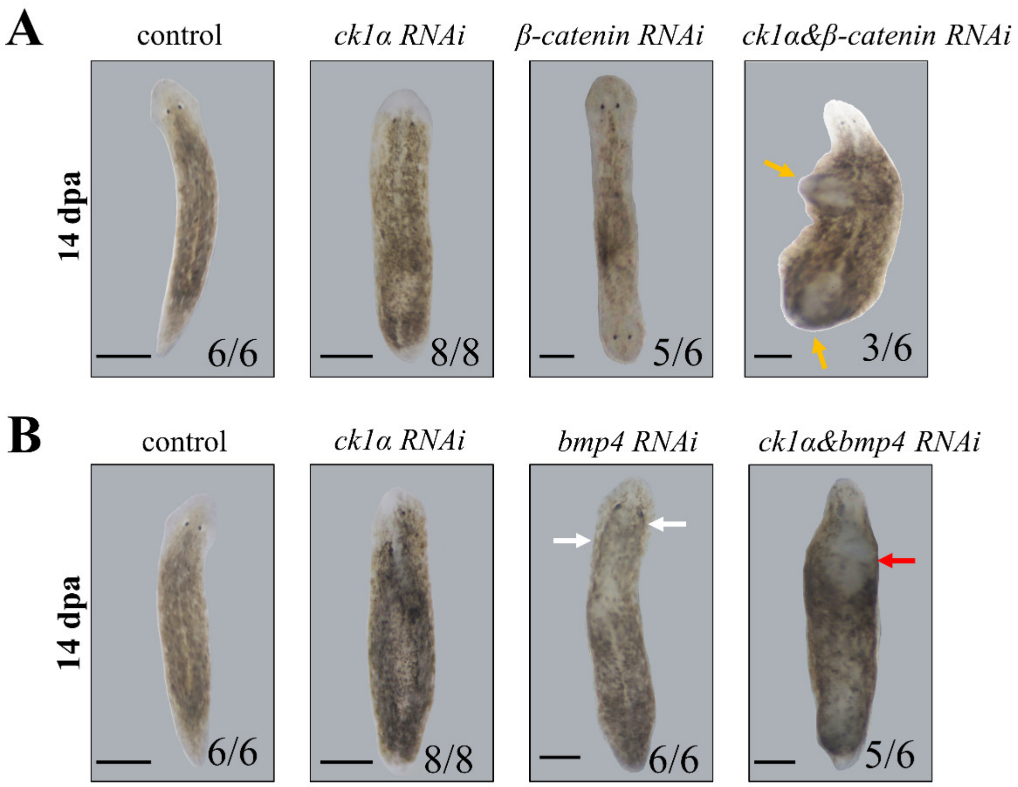

Figure S5. Regeneration defects induced by the double RNAis. Regeneration defects induced by *ck1α&β-catenin* RNAi. Orange arrows: 'outgrowth' in the *ck1α&β-catenin* RNAi animals at 14dpa. (B) Regeneration defects induced by *ck1α&bmp4* RNAi. White arrows: the body appears thicker in *bmp4* RNAi animals. Red arrow: in *ck1α&bmp4* RNAi animals, the body became thicher than *bmp4* RNAi animals. Scale bars: 500 μm.

Table S1. PCR primers used in this study.

**Djck1αF**	ACAAGTGGTAGCAGTAGGTCA
**Djck1αR**	GCCTTAGTCTTCGTATCCTT
**T7-Djck1αF**	TAATACGACTCACTATAGGACAAGTGGTAGCAGTAGGTCA
**T7-Djck1αR**	TAATACGACTCACTATAGGGCCTTAGTCTTCGTATCCTT
**DjslitF**	TCCAGTGTGCAAGTGAAGACA
**DjslitR**	CACACCCTCAAAAGCATCAGC
**T7-DjslitF**	TAATACGACTCACTATAGGTCCAGTGTGCAAGTGAAGACA
**T7-DjslitR**	TAATACGACTCACTATAGGCACACCCTCAAAAGCATCAGC
**Djβ-cateninF**	GCAATATCAACATATGCATCAG
**Djβ-cateninR**	GACATTTGATGAGGTGTAGG
**T7-Djβ-cateninF**	TAATACGACTCACTATAGGGCAATATCAACATATGCATCAG
**T7-Djβ-cateninR**	TAATACGACTCACTATAGGGACATTTGATGAGGTGTAGG
**Djbmp4F**	AATCGACAATTTCCGACACG
**Djbmp4R**	CGTCAACGACAAGAACAACT
**T7- Djbmp4F**	TAATACGACTCACTATAGGAATCGACAATTTCCGACACG
**T7-Djbmp4R**	TAATACGACTCACTATAGGCGTCAACGACAAGAACAACT
**qPCR- Djck1αF**	ACAAGAATCTGACCGGCACC
**qPCR- Djck1αR**	TGAACACGTAGCCCATCGAC
**qPCR-DjslitF**	ACGGGTTTGAGCTCTCTTCC
**qPCR-DjslitR**	TGGTGCTTCGTGAACGGAAT
**qPCR-piwiF**	GAATGAGAGAAGGTCCCAGTTT
**qPCR-piwiR**	GAAGCCGTTCCATCATCATTTG
**qPCR-wnt1F**	GGACGCAGATTTGCGAGAAA
**qPCR-wnt1R**	AACCGCTTGTTCCTTGGCAT
**qPCR-wnt5F**	GGAGGTACTGCCGTGGAAAA
**qPCR-wnt5R**	TTGACACTCCTCGATTCCGA
**qPCR-β-cateninF**	AAGCATGGAGGCCCATTACAT
**qPCR-β-cateninR**	AGCCTCTGGCTAAAATGCGG
**qPCR-bmp4F**	GCACCCCAAAACTATAATGCC
**qPCR-bmp4R**	TAAGGCACACAGCACGGTTT
